# Determining the Relationship between Delivery Parameters and Ablation Distribution for Novel Gel Ethanol Percutaneous Therapy in Ex Vivo Swine Liver

**DOI:** 10.3390/polym16070997

**Published:** 2024-04-05

**Authors:** Erika Chelales, Katriana von Windheim, Arshbir Singh Banipal, Elizabeth Siebeneck, Claire Benham, Corrine A. Nief, Brian Crouch, Jeffrey I. Everitt, Alan Alper Sag, David F. Katz, Nirmala Ramanujam

**Affiliations:** 1 Department of Biomedical Engineering, Duke University, Durham, NC 27708, USA; katriana.von.windheim@duke.edu (K.v.W.); arshbir.banipal@duke.edu (A.S.B.); cnief@stanford.edu (C.A.N.);; 2 Department of Pathology, Duke University Medical Center, Durham, NC 27710, USA; jeffrey.everitt@duke.edu; 3 Department of Radiology, Division of Vascular and Interventional Radiology, Duke University Medical Center, Durham, NC 27710, USA

**Keywords:** ethyl cellulose, ethanol, ablation, low resource, injectable therapy, computed tomography, infusion force

## Abstract

Ethyl cellulose–ethanol (ECE) is emerging as a promising formulation for ablative injections, with more controllable injection distributions than those from traditional liquid ethanol. This study evaluates the influence of salient injection parameters on forces needed for infusion, depot volume, retention, and shape in a large animal model relevant to human applications. Experiments were conducted to investigate how infusion volume (0.5 mL to 2.5 mL), ECE concentration (6% or 12%), needle gauge (22 G or 27 G), and infusion rate (10 mL/h) impacted the force of infusion into air using a load cell. These parameters, with the addition of manual infusion, were investigated to elucidate their influence on depot volume, retention, and shape (aspect ratio), measured using CT imaging, in an ex vivo swine liver model. Force during injection increased significantly for 12% compared to 6% ECE and for 27 G needles compared to 22 G. Force variability increased with higher ECE concentration and smaller needle diameter. As infusion volume increased, 12% ECE achieved superior depot volume compared to 6% ECE. For all infusion volumes, 12% ECE achieved superior retention compared to 6% ECE. Needle gauge and infusion rate had little influence on the observed depot volume or retention; however, the smaller needles resulted in higher variability in depot shape for 12% ECE. These results help us understand the multivariate nature of injection performance, informing injection protocol designs for ablations using gel ethanol and infusion, with volumes relevant to human applications.

## 1. Introduction

Tissue ablation is an inexpensive [1,2] and minimally invasive [3,4,5] alternative to surgical tissue removal for many therapeutic and prophylactic interventions. Ablation typically requires less procedure time [6,7] and shorter hospital stays [7,8] than the surgical approaches. Thermal ablation (e.g., radiofrequency ablation, RFA; microwave ablation, MW; or cryoablation) is common in high-income countries (HICs). Thermal ablation can apply heat or cold: a heating probe can emit high-frequency alternating current (radio frequency ablation, RFA), causing ionic agitation and heat to denature proteins and induce tumor cell necrosis [9,10], or a cryoprobe can induce cold temperatures in tissue which induce intracellular ice crystals that destroy the tumor cells [10,11,12]. However, access to electricity and the cost of supplies significantly limit thermal ablation use in many low- and middle-income countries (LMICs) [13].

Ethanol ablation is a chemical ablation method involving direct injection of ethanol into a lesion to induce coagulative necrosis through protein denaturation [14] and cytoplasmic dehydration [15]. Ethanol ablation is a potentially more suitable global treatment for some cancers, especially superficial solid tumors accessible to injections, since it can be performed by minimally trained personnel in the absence of electricity and/or surgical infrastructure. However, although inexpensive, ethanol is not widely used for tumor ablation. Historically, thermal ablation has produced more predictable and effective outcomes [9]. It has achieved higher rates of complete necrosis and survival for hepatocellular carcinoma treatment as compared to ethanol ablation [16,17,18]. Currently, ethanol ablation often requires multiple injections or treatment sessions [14,19,20,21,22,23,24,25,26,27,28,29], which are not feasible in LMICs, where patients are often lost to follow-up [30]. Moreover, traditional liquid ethanol ablation tends to cause non-target leakage, resulting in irregularly shaped injectate distributions in tumors that typically do not span the entire tumor and may extend to normal cells at the tumor margins [31]. Ethanol volumes that are significantly larger than the corresponding tumor volumes are often needed to achieve efficacy [28]. This exacerbates ethanol leakage into vessels or bile ducts [32], which can result in acute and delayed vascular injuries [33]. Overall, current ethanol ablation methodology results in relatively ineffective treatment, in part due to high interstitial pressure in tumors [34].

Our group previously developed a simple method for tumor ablation by mixing ethyl cellulose (EC), an inexpensive, natural polymer and derivative of cellulose, with ethanol prior to injection. EC increases ethanol viscosity [35] and leverages the endogenous polarity difference between ethanol and water to induce a phase change from liquid to fibrous gel (gelation) upon injection into tissue [36]. This mechanism of in situ gelation sequesters cytotoxic ethanol in the target region, increasing on-target coverage and reducing off-target leakage [37,38].

The physical characteristics and performance of EC depend on its degree of substitution and its molecular weight. EC is obtained through the etherification of cellulose as hydroxyl groups are replaced with ethoxyl groups. At low degrees of substitution, solubility in water can be maintained; however, at higher degrees of substitution EC is only soluble in organic non-polar solvents [39]. When mixed with ethanol, a homogenous sol/gel is formed. This is a slow process that takes between three to twelve hours depending on the concentration of the polymer. The polymer first undergoes a swelling phase that must be continuously mixed to ensure complete dissolution. The viscosity of the solution is dependent on the amount of EC added and has been investigated in previous work [35]. Upon introduction to water, the solution undergoes a phase transition as the ethanol disperses and the EC contacts the poor solvent environment (tissue). This in situ phase change creates a gradient between insoluble EC–fibrous gel–sol/gel as the ethanol diffuses over time, increasing on-target coverage and reducing off-target leakage [37,38]. The speed and degree to which the phase change occur depend on the initial concentration of EC [35]. Higher ECE concentrations provide better retainment of ethanol as they generate more of a gel, but the higher viscosity creates a tradeoff between increasing efficacy and decreasing injectability. EC has been highlighted as a promising cellulose derivative for modified drug delivery due to these properties and has been implemented in pharmaceutical research [40].

We previously showed that ECE ablation induced complete regression of squamous cell carcinomas in a hamster cheek pouch model, while conventional ethanol ablation did not induce regression [35]. Injected ECE also exhibited decreased localized adverse events and increased overall survival in a syngeneic model of breast cancer, demonstrating the increased safety and efficacy of ECE in small animal models compared to ethanol alone [41]. Finally, injected ECE reduced tumor volume and was demonstrated as a feasible treatment for feline squamous cell carcinoma [42]. Overall, injected ECE ablation has the potential to address the need for a low-cost ablative therapeutic for the treatment of cancer in LMICs. Further, HICs could benefit from non-thermal ablation methods to treat tumors in locations not amenable to thermal ablation [43], or due to patient exclusion criteria, preference, and/or cost.

Ideally, any clinically suitable ablation technique should achieve predictable and repeatable injection distribution volumes in humans. Those volumes should span and ablate the target tumor volume, while minimizing off-target toxicity. In RFA, the temperature decreases with distance from the source; only the area within a lethal temperature threshold is destroyed [15,19,21]. Clinical data for thermal ablations have been used to generate charts that show an effective ablation diameter, height, or width vs. various procedure settings (i.e., temperature, ablation time, number of probes). These charts guide clinical practice. Analogously, an understanding of the influence of key injection parameters on ECE ablation will be important for its clinical translation.

The goal of the present study was to help inform an analogous approach to optimizing ECE injections, as has been created for thermal ablations. We specifically focused upon the formulation of the therapeutic ECE (viz. ethanol concentration) and key injection parameters in relation to the resulting force and distribution volumes for clinically relevant (less than 5 cm in diameter as is typical for ablative therapies) tumor treatment [2]. This study followed up our previous work, which developed methodologies to relate key injection parameters to injected ECE injection volumes (50–200 µL) in ex vivo swine and rat liver [37,38]. We also investigated the force required for infusion to inform the impact that the high viscosity of ECE will have on the safety and efficacy of these injections. The force during injection has previously been tied to pressure during injection. However, the literature is limited on substances with viscosities as high as ECE’s [44]. Higher forces during injection correlate with higher pressures in the tissue, leading to tissue cracking, leakage, and lower retainment in the zone of ablation [37]. Typically, clinical thermal ablation devices, such as RFA, treat lesions that are 1 to 5 cm in diameter, corresponding to a spherical volume ranging from approximately 0.52 mL to 65.45 mL [14,16,17,18,21,23,24,25,27]. Our prior work focused upon smaller volumes (50–200 µL). Scaling up injection volume is a complex biophysical process, where injection variables (e.g., volume, rate, needle gauge, EC concentration) are likely to be salient and do not scale up obviously. The work here addressed this problem.

Our previous preclinical studies with small injection volumes (50–200 µL) considered swine liver, as well as ex vivo and in vivo rat liver tissue models [37,38]. In these studies, we found that for small infusion volumes (50–200 µL), 6% ECE significantly increased depot volume compared to pure ethanol and 3% ECE at infusion rates of 10 mL/h in ex vivo swine liver [37]. We also found in ex vivo rat liver that 12% ECE, vs. lower ECE concentrations and pure ethanol, significantly increased ethanol spatial distribution and that there was a strong radiologic–pathologic correlation between ethanol distribution volumes observed on CT imaging and resultant necrosis [38]. We then followed this work up with an in vivo rat liver model and demonstrated superior ethanol spatial distribution of 12% ECE compared to pure ethanol in vivo, thus confirming the ex vivo results [38]. The present study builds upon the foundational knowledge of our previous preclinical work, to investigate injection volumes relevant to human applications. In so doing, we also began a more in-depth analysis of biophysical factors governing the injection process, focusing upon the force required to push the formulation through the needle.

## 2. Materials and Methods

### 2.1. Formulation of ECE

For this study, the EC obtained from Sigma-Aldrich (St. Louis, MO, USA) had a viscosity of 100 cP and a 48% ethoxyl content. This corresponds to a degree of substitution of 2.35–2.62 [45]. The molecular weight of 100 cP EC is characterized as 80.8 ± 24 kDa [46]. Ethanol 200 proof (anhydrous ethanol, Koptec, King of Prussia, PA, USA) was mixed with EC within 24 h of the experiment.

### 2.2. Assessment of Distribution Volume of ECE In Ex Vivo and In Vivo Swine Liver

Previous work in our lab used ex vivo swine liver, as well as ex vivo and in vivo rat liver tissue models [37,38]. However, the infusion volume in all of these studies was 50–200 µL, which is quite small compared to lesion sizes that are typically treated with ablation in humans. The swine liver is an appropriate model choice for ablation assessment. While the swine liver differs from the human liver in external morphology, it has a similar size, segmental anatomy, vascularity, and biliary tree structure [47]. The efficacy of ablative therapies is commonly examined and in healthy liver tissue [48,49,50,51,52,53,54,55,56] specifically, traditional ethanol ablation [32,48,57] (PEI), radiofrequency ablation [54,58] (RFA), microwave ablation [55,59,60,61] (MWA), drug-eluting bead embolization [62], and high-intensity focused ultrasound [63] (HIFU) are used. These relatively homogeneous tissues provide a consistent environment for such studies. Further, ex vivo liver is commonly used by medical device companies to create guidelines for ablation parameter choices to achieve ablation zones of varying sizes. Therefore, porcine liver was an appropriate choice here for assessment of ECE ablation distribution for clinically relevant volumes.

### 2.3. Experimental Design

We scaled up previous work using small volumes in ex vivo and in vivo rat livers to investigate infusion parameters for ECE ablation to achieve distribution volumes of a clinically relevant size. Since percutaneous ablation is most often used for, and most successful in, the treatment of smaller tumors [15,19,21,22,25], we investigated parameters to achieve ablation volumes in the range of 0–1.5 mL, since a sphere with a radius of 1 cm corresponds to an approximately 0.5 mL volume. Using the methods established in prior work [38], we conducted injections of ECE while varying the parameters of formulation (6, 12% ECE), infusion volume (0.5, 1, 1.5, 2, 2.5 mL), needle gauge (22, 27 G), and infusion rate (10 mL/h, manual infusion). Amounts of 6% and 12% were the most commonly used EC concentrations in our previous studies [37,38]. Parameters were systemically varied in a series of three experiments, selectively reducing the parameter space from a full factorial design. The completed experiments are detailed in Table 1. Experiment (Exp.) 1 was designed to establish an optimal ECE concentration, with the goal of maximizing distribution volume in the tissue to reduce the amount and cost of the formulation. Thereafter, we kept the ECE concentrations constant. Exp. 2 was designed to examine the effects of using a larger needle, which would result in reduced pressure during injection and is closer to commonly used clinical needle and probe sizes for current ablation methods [9,10,14,27,28,64,65,66]. Finally, Exp. 3 focused upon the effect of infusion rate on resultant distribution volume. We included manual infusions, which would enable easier clinical translation, greater physician flexibility, and reduced need for costly injection equipment. For Exp. 3, smaller infusion volumes were investigated, since they achieved superior ratios of distribution volumes to infusion volumes in previous experiments. Both needle gauges were studied. Notably, the smaller needle would enhance patient comfort [38].

### 2.4. Experimental Procedure

Ablations in ex vivo swine livers, acquired from Hatley Farms or Animal Biotech Industries, were completed using the respective conditions detailed in Table 1. Upon delivery, the swine livers were immediately preserved and stored in Krebs–Ringer bicarbonate buffer (Sigma-Aldrich, K4002) on ice until injection (within 1–2 h). For injections, the livers were submerged in the Krebs–Ringer bicarbonate buffer in a gallon plastic zip bag which contained no air bubbles to promote higher contrast in imaging. ECE was infused from a 3 mL syringe (BD Medical, Columbus, NE, USA) through 10 cm of rubber tubing (1/4 in. inner diameter; McMaster-Carr, Douglasville, GA, USA), using a syringe pump (NE-1000; New Era, Farmingdale, NY, USA). The needle was inserted directly through the plastic zip bag and into the liver. In all experiments, needles were inserted to a depth 0.5 inches below the surface of the liver. 

Pre- and post-ablation computed tomography (CT) images were acquired using a GE Discovery 690 PET/CT Scanner (General Electric, Waukesha, WI, USA) and segmented using 3D Slicer software [67] version 4.11.20210226 r29738/7a593c8 (minimum of *n* = 6 per group). In each imaging session, ethanol–water solutions with 0%, 25%, 50%, 75%, and 100% ethanol concentrations were prepared to assess ethanol CT contrast and to create the calibration equation (established in our prior studies [38]). This enabled us to quantify a 20% ethanol concentration cutoff value for ablation segmentation. Radiodensity values corresponding to at least 20% ethanol concentration [68] were included in the segmentation of ECE. To help elucidate the effect of infusion parameters (infusion volume, EC concentration, needle gauge, and infusion rate) on the resultant ablation, we quantified the ablation metrics: the depot volume (distribution volume of the injectate without inclusion of non-depot injection spread into nearby collapsed vasculature), percent of infusion volume retained in the depot, and aspect ratio of the depot.

### 2.5. Computed Tomography Image Acquisition

All CT images were acquired on a GE Discovery 690 PET/CT, obtained using a helical scan with a 1 sec rotation time, full rotation length, 20 mm detector coverage, and 0.625 mm helical thickness. The pitch and speed (mm/rot) were 0.969:1 and 19.37, respectively. A medium body single field of view (SFOV) was used with 120 kV and auto/smart mA. For reconstruction, a 36 cm field of view (FOV) was used with Standard Plus reconstruction and no adaptive statistical iterative reconstruction (ASiR) Setup.

### 2.6. Image Segmentation in 3D Slicer

Images were processed in 3D Slicer [67]. The radio threshold for ethanol segmentation was determined using the radiodensities of ethanol–water vials. The average radiodensity of in vitro ethanol–water samples was computed by segmentation of a sphere within each vial. Specifically, spheres with diameters of 30 mm were created in the Segment Editor module and centered in each vial. Smaller spheres were used if the 30 mm diameter sphere was larger than the liquid volume in the vial. The Segment Statistics module was used to calculate the mean radiodensity in each vial.

Ex vivo liver images were segmented by selecting the tissue surrounding the injected ethanol distribution without including surrounding buffer. Ethanol was segmented using the 20% cutoff value determined from standard vials imaged at each imaging session, as detailed in prior work [38]. Specifically, segmentations were made in the Segment Editor modules by using a sphere brush to paint around the injection site through all image slices, excluding areas outside the liver. This segment was named the “true volume” of the ethanol distribution and is depicted in Figure S1A, for 1 mL infusions of both 12% ECE and 6% ECE, using 27 G and 22 G needles. The collapsed nature of the vasculature in the ex vivo swine liver leads to a low-resistance path for flow of the injectate. The true volume includes non-depot injection spread into nearby collapsed vasculature. This is not representative of the ablation zone, since in an in vivo system, such a spread would be cleared away from the target by the vascular system. The true volume segmentation was duplicated into a new segmentation and any portions of the segmentation not in the depot (the ablation region at the target) were removed. The new segment was named the “depot volume” and is depicted in Figure S1B as the “segmented depot”. The depot volume does not include any non-depot injection spread into nearby collapsed vasculature. The Segment Statistics module was used to calculate the volume of both the true volume and depot volume distributions in the tissue. The Screen Capture module was used to export the segmentation volumes as videos and images. Segmentation and cross-sectional images were generated directly from 3D Slicer; analysis of segmentations and generation of histograms was performed in MATLAB R2022a. Statistical analysis was completed in JMP Software (JMP^®^, Version 17, 1989–2023; SAS Institute Inc., Cary, NC, USA).

### 2.7. Force Measurements

All force measurements were conducted with a calibrated load cell (Loadstar Sensors, Fremont, CA, USA) connected to a DI-1000 interface obtained through McMaster-Carr (Elmhurst, IL, USA). All injections were conducted into air. The configuration for these injections was the same as for the ex vivo swine liver injections, except that the tubing between the syringe and needle was omitted. Table 1 gives the parameter values tested, except manual measurements that were also performed. The load cell was placed at the back of the syringe plunger to record the force required to expel the ECE. Force was recorded every 1 s using LoadVUE Pro software v2021.8.20.1 (Loadstar Sensors, Fremont, CA, USA). The load cell was zeroed before each measurement, and the syringe pump base was placed a few millimeters away from the syringe plunger for each injection to capture the break loose force. When the desired injection volume was reached, the movement in the syringe pump was halted, and the pressure was allowed to drop off for a few seconds before the force-recording software was stopped. Force was measured every second, and thus, the number of data points varied with the infusion volume (*n* = 180 for 0.5 mL, *n* = 900 for 2.5 mL). For data analysis, the injection force profiles were summarized by averaging two time points, an initial one at 100 s and a final one at 50 s, prior to the end of the injection.

### 2.8. Selection of Injection Parameters

Previous studies in our group investigated different combinations of ECE concentrations, needle gauges, and volumes. These selections were motivated by experimental questions and model limitations (i.e., tumor size limiting volume delivered). While these studies were critical for establishing the potential efficacy of the treatment, the variety of parameters chosen did not lead to an algorithm for determining the best injection parameters. The work here contributes to the development of this algorithm, by combining information on injection parameters gained in our previous studies with new knowledge about how parameter choice impacts the amount of ECE that is maintained in the depot. The goal was to maximize the amount of ECE that is retained while operating within clinical guidelines. Except for volume, which was increased to clinically relevant volumes, all injection parameters were selected from our previous studies. The needle gauges 22 and 27 were also what we previously studied.

### 2.9. Statistical Analyses

All statistical analysis was conducted in JMP. The force data were tested for unequal variances (Levene’s test) and analyzed with a general linear models methodology to assess the impact of ECE concentration, needle gauge, and volume on the in vitro injection force. In vivo experiments were analyzed with multi-factor ANOVA to assess the impact of ECE concentration, needle gauge, and volume on depot size, percent retained, and aspect ratio. A significance level of *p* = 0.05 was applied throughout.

## 3. Results

### 3.1. Force of Injection Increases with Higher ECE Concentration and Smaller Needle Gauge

We investigated the force required for the injection of 6% and 12% ECE through 22 and 27 G needles at a 10 mL/h flow rate into air (called the “infusion force”). The volumes used were matched to the swine liver experiments detailed in Table 1. The flow rate was held constant for all injections; thus, infusion time varied depending on injection volume. 

**Figure 1:**
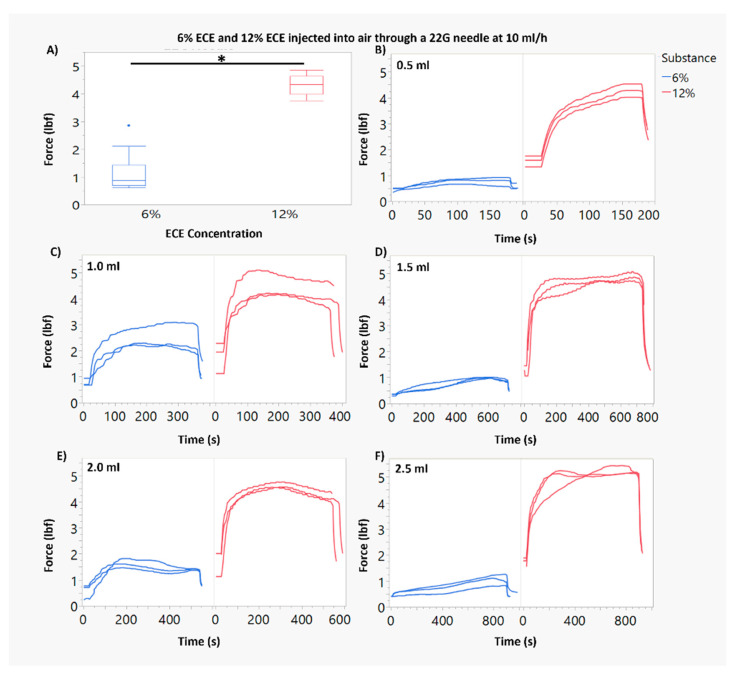
Comparison of force during injection for 6% and 12% ECE through 22 G needles. (**A**) Force measurements for 6% ECE and 12% ECE through a 22 G needle at 10 mL/h. The force required for 12% ECE was significantly larger than the force for 6% (* *p* < 0.05). (**B**–**F**) Plots of the force measured over time during an infusion for 0.5–2.5 mL infusions through a 22 G needle at 10 mL/h.

**Figure 2:**
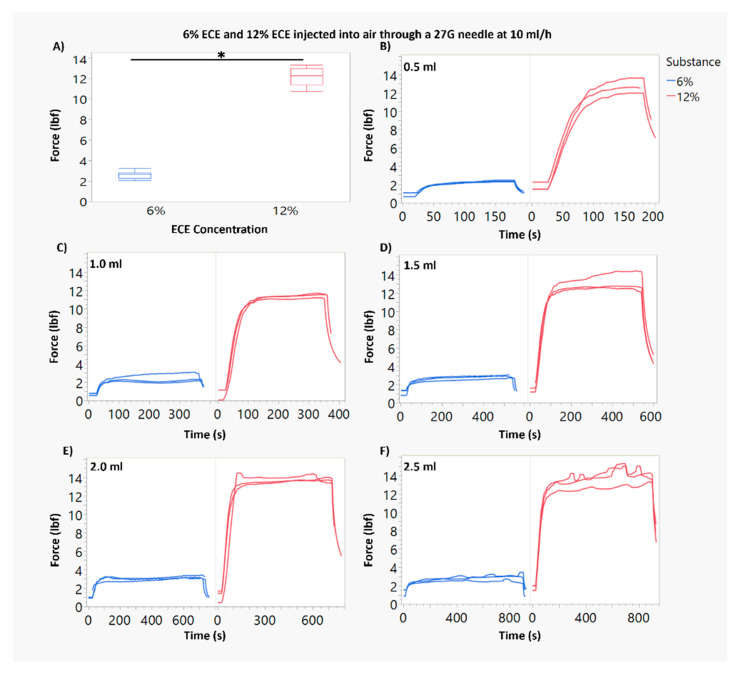
Comparison of force during injection for 6% and 12% ECE through 27 G needles. (**A**) Force measurements for 6% ECE and 12% ECE through a 27 G needle at 10 mL/h. The force required for 12% ECE was significantly higher than 6% ECE (* *p* < 0.05). (**B**–**F**) Plots of the force measured over time during an infusion for 0.5–2.5 mL infusions through a 27 G needle at 10 mL/h.

Figure 1A and Figure 2A show box plots for the average force of injection for each ECE concentration and needle gauge combination. In Figure 1B–F, the force profiles vs. time are shown for all injections (*n* = 3) through 22 G needles. Figure 2B–F show the force profiles vs. time for all injections (*n* = 3) through 27 G needles. Approximately 90 s lapsed before the force plateau during an injection. 

By applying the Hagen–Poiseuille equation (Hagen–Poiseuille, 1846) to our injections, volume should have had no impact on force for injections to air. Therefore, using multiple volumes was not theoretically critical. However, by using multiple volumes, we were able to further ensure that the force measurement methodology was working properly. Volume was found to only be significant (*p* < 0.05) when the 0.5 mL volume was included as a group. This is theorized to be due to the long rise in force required for the ECE injections to reach the plateau. The 0.5 mL infusion is small enough that the time spent at plateau is very short, putting it on the lower end of force measurements. With this in mind, volume was collapsed as a factor to elucidate the statistical power of the remaining factors, ECE concentration and needle gauge. The injection force ranged from an average (over replicates) of 1.19 lbf for 6% ECE through 22 G needles, to an average of 12.2 lbf for 12% ECE through 27 G needles (Figure 1A and Figure 2A).

For each ECE concentration (6% and 12% ECE), needle gauge had a significant impact on the force required for infusion. The 27 G needle required approximately a three-fold increased infusion force compared to the 22 G needle, per ECE concentration. ECE concentration also had a significant impact on force. For the 27 G needle, 12% ECE required approximately a three-fold increased force compared to 6% ECE. For the 22 G needle, 12% ECE required approximately a five-fold higher force compared to 6% ECE. 

Additionally, we observed increased variability in the required force for infusion when using a higher ECE concentration and smaller needle gauge. This can be noted visibly in Figure 2F. Statistically, Levene’s test confirmed that variances were significantly different between both ECE concentrations and needle gauges, but not across the volumes.

### 3.2. Increasing Infusion Volume of 12% ECE Achieves Significantly Greater Treatment Volumes, While Increasing the Infusion Volume 6% ECE Does Not Yield a Significant Increase in Treatment Volume, for Both 22 G and 27 G Needles

As detailed in Table 1, Exp. 1 aimed to examine the influence of EC concentration on the resultant distribution volume and distribution shape for clinically relevant ablation volumes (0.5–2.5 mL) using a 27 G needle. The relevant treatment volume is referred to as the “depot volume”, which is defined as the distribution of the injectate within the tissue, excluding any leakage of the injectate into nearby collapsed vasculature as a result of the ex vivo model (Figure S1). All infusions were performed using 27 G needles and an infusion rate of 10 mL/h, as was used in previous preclinical studies in mice [41,69,70].

**Figure 3:**
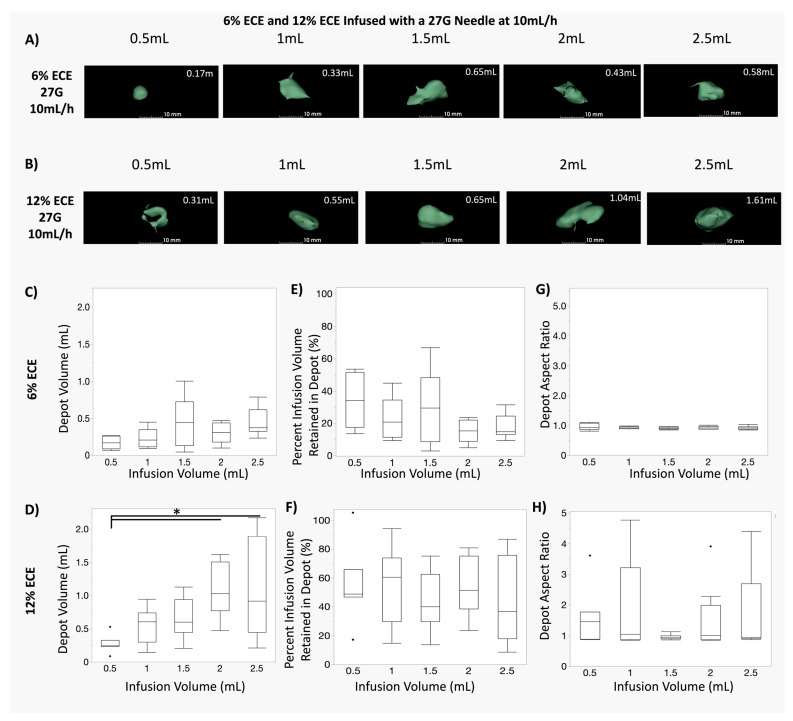
Comparison of infusions of 6% and 12% ECE through a 27 G needle. (**A**,**B**) Representative 3D segmentations of the depot volume for 0.5 mL, 1 mL, 1.5 mL, 2 mL, and 2.5 mL infusion volumes for 6% ECE (**A**) and 12% ECE (**B**) using 27 G needles and a 10 mL/h infusion rate. (**C**,**D**) Box plots showing the resultant depot volume achieved at various (0.5–2.5 mL) infusion volumes of 6% ECE (**C**) and 12% ECE (**D**) using 27 G needles and a 10 mL/h infusion rate (* *p* < 0.05). No significant differences were observed between any of the infusion volumes (0.5–2.5 mL) for 6% ECE. However, both 2 mL and 2.5 mL infusion volumes achieved significantly larger distribution volumes than the 0.5 mL infusion volume for 12% ECE. (**E**,**F**) Box plots showing the percent infusion volume retained in the depot for various infusion volumes (0.5–2.5 mL) of 6% ECE (**E**) and 12% ECE (**F**) using 27 G needles and a 10 mL/h infusion rate. Significant differences were not observed for either 12% ECE or 6% ECE. (**G**,**H**) Box plots of the aspect ratio achieved at various infusion volumes (0.5–2.5 mL) for 6% ECE (**G**) and 12% ECE (**H**) using 27 G needles and a 10 mL/h infusion rate. While no significant differences in the aspect ratio were found, there was a larger range of aspect ratios for 12% ECE compared to 6% ECE.

Figure 3A,B show representative images of the depot volumes for 0.5 mL, 1 mL, 1.5 mL, 2 mL, and 2.5 mL infusion volumes for both 6% ECE (A) and 12% ECE (B). The quantified depot volume is printed in white in the top right of each image. Figure S2 shows the corresponding representative images of the non-depot volumes prior to segmentation for 0.5 mL, 1 mL, 1.5 mL, 2 mL, and 2.5 mL infusion volumes for both 6% ECE (Figure S2A) and 12% ECE (Figure S2B).

Figure 3C,D show box plots for the depot volume achieved at various infusion volumes, for concentrations of 6% ECE (Figure 3C) and 12% ECE (Figure 3D). No significant differences in depot volume were observed between any of the infusion volumes (0.5–2.5 mL) for 6% ECE. However, for 12% ECE, both 2 mL and 2.5 mL infusion volumes achieved significantly larger depot volumes than the 0.5 mL infusion volume (*p* < 0.05). Increasing the infusion volume significantly increases the treatment volume when 12% ECE is used, but not 6% ECE.

Figure 3E,F show box plots for the percent infusion volume retained in the depot for various infusion volumes for 6% ECE (Figure 3E) and 12% ECE (Figure 3F). Significant differences were not observed for either 6% ECE or 12% ECE, indicating that the infusion volume did not significantly influence the percent of the infusion volume retained in the depot for either polymer concentration. Figure 3G,H show box plots for the depot aspect ratio achieved at various infusion volumes for 6% ECE and 12% ECE, respectively. Again, no significant differences were observed for either 6% ECE or 12% ECE, indicating that the infusion volume does not significantly influence the shape of the distribution in tissue. However, it was observed that there was a wider range of aspect ratios for 12% ECE compared to 6% ECE. That is, the lower polymer concentration was more effective at achieving a predictable distribution shape. It is possible that this was due to the larger forces generated when using 12% ECE compared to 6% ECE (Figure 1 and Figure 2). The mechanics of tissue deformation, and possible localized rupture due to these increased forces, may have produced more irregularly shaped distributions in the tissue. Interestingly, after segmenting leakage into nearby vessels for both polymer concentrations, the median aspect ratio was near 1 for all infusion volumes. That is, the shape of the depot volume (without any leakage included) of these large volume infusions (0.5–2.5 mL) was relatively spherical. Spherical ablation zones allow clinicians improved prediction of treatment effect effects; they are common for existing clinically used modalities such as microwave or radiofrequency ablation and cryoablation.

**Figure 4:**
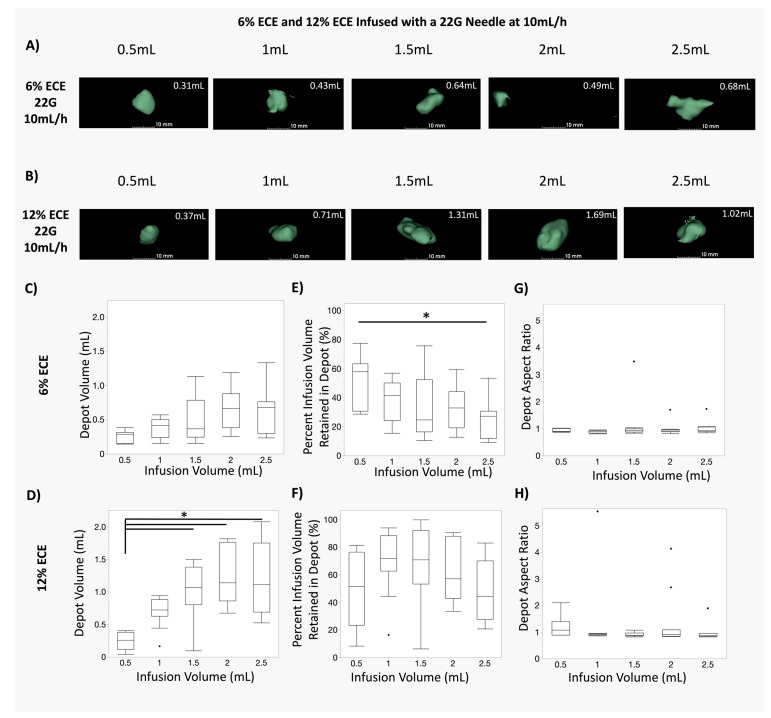
Comparison of infusions of 6% and 12%ECE through a 22 G needle. (**A**,**B**) Representative 3D segmentations of the depot volume for 0.5 mL, 1 mL, 1.5 mL, 2 mL, and 2.5 mL infusion volumes for 6% ECE (**A**) and 12% ECE (**B**) using 22 G needles and a 10 mL/h infusion rate. (**C**,**D**) Box plots showing the resultant depot volume achieved at various (0.5–2.5 mL) infusion volumes of 6% ECE (**C**) and 12% ECE (**D**) using 22 G needles and a 10 mL/h infusion rate (* *p* < 0.05). No significant differences were observed between any of the infusion volumes (0.5–2.5 mL) for 6% ECE. However, 1.5 mL, 2 mL, and 2.5 mL infusion volumes achieved significantly larger distribution volumes than the 0.5 mL infusion volume for 12% ECE. (**E**,**F**) Box plots showing the percent infusion volume retained in the depot for various infusion volumes (0.5–2.5 mL) of 6% ECE (**E**) and 12% ECE (**F**) using 22 G needles and a 10 mL/h infusion rate (* *p* < 0.05). A significantly lower percent of the infusion volume was retained in the depot for 2.5 mL compared to 0.5 mL infusions for 6% ECE. Significant differences were not observed for 12% ECE. (**G**,**H**) Box plots of the aspect ratio achieved at various infusion volumes (0.5–2.5 mL) for 6% ECE (**G**) and 12% ECE (**H**) using 22 G needles and a 10 mL/h infusion rate. No significant differences in aspect ratio were found for 12% ECE or 6% ECE.

We then examined how the same injection parameters’ (infusion volume, EC concentration) influence on the depot volume, injection volume retention, and depot shape using a more clinically relevant needle size, the 22-gauge needle (Exp. 2). This size is appropriate for human applications, but too large for humane use in small animals. The results of Exp. 2 are shown in box plots in Figure 4. Figure 4A,B show representative images of the depot volumes for various infusion volumes for both 6% ECE and 12% ECE, respectively. The quantified depot volume is printed in white in the top right of each image. Figure S2 shows corresponding representative images of the non-depot volumes prior to segmentation for both 6% ECE (C) and 12% ECE (D) for the 22 G needle.

Figure 4C,D show box plots for the resultant depot volume at various infusion volumes, for ECE concentrations of 6% ECE (Figure 4C) and 12% ECE (Figure 4D). No significant differences in depot volume were found between any of the infusion volumes for 6% ECE using a 22 G needle, consistent with results for 6% ECE using a 27 G needle (Figure 3C). However, 1.5 mL, 2 mL, and 2.5 mL infusion volumes achieved significantly larger depot volumes than the 0.5 mL infusion volume for 12% ECE (*p* < 0.05), consistent with the depot volume results for 12% ECE using a 27 G needle (Figure 3D). Thus, increasing the infusion volume significantly increased the treatment volume when 12% ECE was used for both 27 G and 22 G needles, but not 6% EC. Taken together, the results in Figure 3 and Figure 4 suggest that the higher polymer concentration could be more effective at maximizing the depot volume, regardless of the needle size.

Figure 4E,F show box plots for the percent infusion volume retained in the depot in the 22 G needle for 6% ECE and 12% ECE, respectively. The 6% ECE showed a significant decrease in percent retained for 2.5 mL infusions compared to 0.5 mL infusions (*p* < 0.05), indicating that increasing the infusion volume of 6% ECE with a 22 G impairs improved injection coverage. No significant differences were observed for 12% ECE, indicating that injection coverage was relatively stable across volumes. Finally, Figure 4G,H show box plots for the depot aspect ratio achieved at various infusion volumes for 6% ECE and 12% ECE, respectively. No significant differences were observed for either 6% ECE or 12% ECE, indicating that the infusion volume did not have a significant influence over the shape of the distribution in tissue. Interestingly, the larger range of aspect ratios for 12% ECE and the 27 G needle (Figure 3H) was not observed for the 12% ECE with the 22 G needle. These results indicate that the combination of the small 27 G needle gauge and a higher polymer concentration may be less effective at achieving reliable and predictable distribution shapes. Finally, for all experimental groups, the median aspect ratio was near 1 when using the 22 G needle, indicating a relatively spherical ablation shape, consistent with results from Exp. 1 using the 27 G needle (Figure 3).

### 3.3. At Smaller Infusion Volumes, 12% ECE and 6% ECE Achieve Similar Treatment Volumes, but 12% Achieves Superior Treatment Volumes and Higher Percent Retained at the Target Site as Infusion Volume Increases Compared to 6% ECE, Regardless of Infusion Volume or Needle Gauge

**Figure 5:**
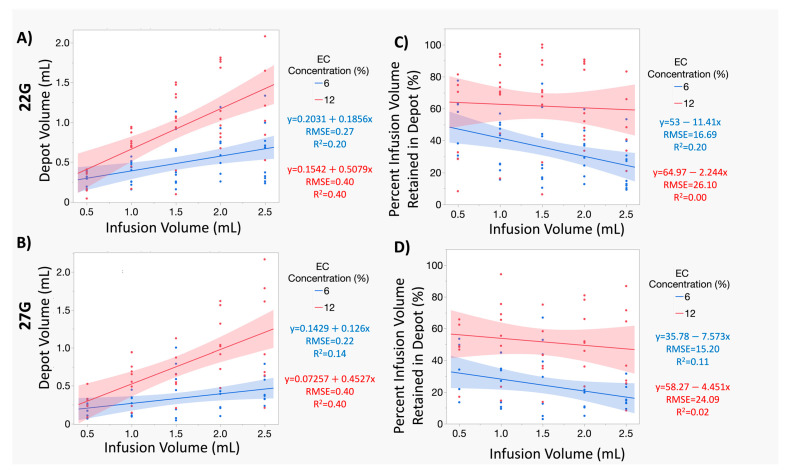
Trends in the relationship between depot volume and percent infusion volume retained in the depot to the infusion volume. (**A**,**B**) Line plots of the relationship between the depot volume and infusion volume for infusions performed with 22 G (**A**) and 27 G (**B**) needles. For the 22 G needle (**A**), the 6% ECE (*p* < 0.05, r^2^ = 0.24) and 12% ECE (*p* < 0.05, r^2^ = 0.40) both show positive relationships between depot volume and infusion volume, with the 12% ECE achieving a stronger correlation between depot volume and infusion volume. Similar to the 22 G, for the 27 G needle (**B**), the 6% ECE (*p* < 0.05, r^2^ = 0.14) and 12% ECE (*p* < 0.05, r^2^ = 0.40) both show positive relationships between depot volume and infusion volume, with the 12% ECE achieving a stronger correlation between depot volume and infusion volume. (**C**,**D**) Line plots of the relationship between the percent of the infusion volume retained in the depot and infusion volume for infusions performed with 22 G (**A**) and 27 G (**B**) needles. For the 22 G needle (**C**), the 6% ECE (*p* < 0.05, r^2^ = 0.20) shows a negative relationship between the percent retained and the infusion volume. The 12% ECE (*p* < 0.05, r^2^ = 0.00) shows a more stable percent retained as infusion volume increases. Similar to the 22 G, for the 27 G needle (**D**), the 6% ECE (*p* < 0.05, r^2^ = 0.11) shows a slightly negative relationship between the percent retained and the infusion volume. The 12% ECE (*p* < 0.05, r^2^ = 0.02) shows a similar slight negative relationship between the percent retained and the infusion volume, but with greater retention at all volumes compared to the 6% ECE.

Next, we sought to understand relationship trends amongst infusion volume, depot volume, and percent retained for the various injection parameters explored. While Figure 3 and Figure 4 allow us to examine the impact of increasing infusion volume at set intervals, they do not allow us to easily compare the trends in the relationships of our independent and dependent variables or investigate the influence of a needle gauge within a set EC concentration. Figure 5 shows line plots and confidence intervals between the infusion volume vs. resultant depot volume, and the percent infusion volume retained in the depot, respectively, for both 6% ECE (blue) and 12% ECE (red). These plots are not meant to be used for predictive modeling, but rather to illuminate trends in the overall relationships of these injection parameters. Figure 5A,B show a positive, significant (albeit weak) correlation between infusion and depot volume, regardless of polymer concentration (*p* < 0.05 for both 6% and 12% ECE), and for both the 22 G and 27 G needles. Regardless of the needle gauge, 12% ECE trends toward a higher positive slope (22 G: 0.5709; 27 G: 0.4527) than 6% ECE (22 G: 0.1856; 27 G: 0.1260) and a higher, albeit low, $r^{2}$ value (22 G: 0.40 vs. 0.20; 27 G 0.40 vs. 0.14). These trends cannot be accurately used for predictive modeling but are insightful to the overall relationships between these parameters. Slopes for the 22 G and 27 G needle were similar, regardless of EC concentration, indicating that when the polymer concentration was constant, the influence of the needle gauge on the resultant depot volume was negligible. The stronger correlation between the infusion volume and resultant depot volume for 12% ECE indicates that it is easier to maximize the depot volume by increasing the infusion volume for 12% ECE compared to 6% ECE, regardless of the needle gauge used for infusion. There is significant overlap in the 6% ECE and 12% ECE curves for 0.5 mL and greater separation between the curves as infusion volume increased for both needle gauges investigated. Thus, at the smaller infusion volumes, the impact of EC concentration on depot size was minimal. For smaller target volumes, the tradeoff in EC concentration is negligible, and resources could be conserved by using lower concentrations.

Figure 5C,D show trends for negative correlations between infusion and percent infusion volume retained in the depot, regardless of polymer concentration (*p* < 0.05 for both 6% and 12% ECE) for both the 22 G (Figure 5C) and 27 G (Figure 5D) needles. This negative correlation is likely due to the leakage of the ECE into nearby vessels (the non-depot volume), due to the high prevalence of collapsed vasculature in the ex vivo swine liver. For larger injections, there is a larger potential for leakage as the probability of the depot expanding to hit nearby vasculature is increased. Regardless of the needle gauge, the 12% ECE trended toward a more positive slope (22 G: −2.244; 27 G: −4.451) than 6% ECE (22 G: −11.41; 27 G: −7.573), indicating that retention of the injection at the target site was more consistent regardless of the volume infused for 12% ECE. The percent retained for the smallest infusion volume (0.5 mL) for the 22 G needle infusions was more similar for the 6% ECE and 12% ECE, than when a 27 G needle was used. We hypothesize that this difference is due to the reduced pressure when a 22 G needle is used (Figure 1), which may reduce crack formation. However, when infusion volume is increased, this increases the overall pressure (Figure 1), enough to result in leakage and reduced retention.

Notably, the slopes for the 22 G and 27 G needles appeared comparable when the EC concentration was constant, indicating negligible influence of the needle gauge. However, while retention remained consistent regardless of infusion volume for 12% ECE, retention decreased with increasing infusion volume for 6% ECE. To improve retention at the injection site, a higher EC concentration or reduced infusion volume may be necessary, but may not always be practical due to injection pressure or resource limitations.

### 3.4. The Impact of the Infusion Rate Is Not Significant for Smaller (0.5–1 mL) Infusion Volumes of 12% ECE for Both 22 G and 27 G Needles

**Figure 6:**
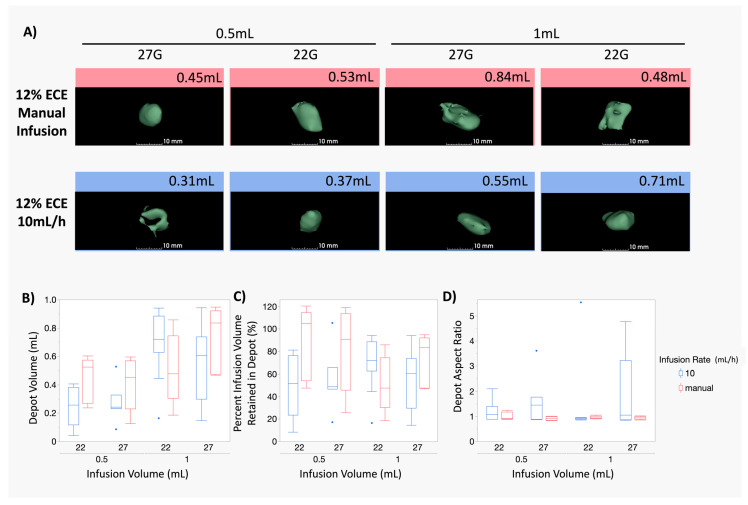
Comparison of manual infusion and 10 mL/h infusion rates for 0.5 mL and 1 mL infusions of 12% ECE. (**A**) Representative 3D segmentations of the depot volume for 0.5 mL and 1 mL infusion volumes for 12% ECE using both 22 G and 27 G needles and 10 mL/h and manual infusion rates. (**B**) Box plots showing the resultant depot volumes achieved at small infusion volumes (0.5–1 mL) of 12% ECE for both 22 G and 27 G needles at both 10 mL/h (blue) and manual (red) infusion rates. No significant differences were observed. (**C**) Box plots showing the percent infusion volume retained in the depot for at small infusion volumes (0.5–1 mL) of 12% ECE for both 22 G and 27 G needles at both 10 mL/h (blue) and manual (red) infusion rates. No significant differences were observed. (**D**) Box plots showing the aspect ratio at small infusion volumes (0.5–1 mL) of 12% ECE for both 22 G and 27 G needles at both 10 mL/h (blue) and manual (red) infusion rates. No significant differences were observed.

Finally, Exp. 3 aimed to examine the influence of a manual infusion rate for the smaller infusion volumes (0.5 mL and 1 mL). Figure 6A shows representative images of the 12% ECE ablations performed using 27 G and 22 G with either manual (red outline) or 10 mL/h (blue outline) infusion rates. Figure S3 shows the corresponding representative images of the non-depot volumes prior to segmentation for 0.5 mL and 1 mL infusion volumes for both 6% ECE and 12% ECE and both 22 G and 27 G needles.

Figure 6B compares the depot volume achieved using a 10 mL/h infusion rate (blue) and manual infusion (red). Interestingly, no significant differences were observed between the depot volume achieved, regardless of infusion volume or needle gauge, consistent with the results of Figure 5; needle gauge does not significantly influence ablation depot volume or retention. Further, these results indicate that for smaller ablations (0.5–1 mL), the infusion rate has no significant influence on the resultant depot volume.

Figure 6C shows box plots for the percent infusion volume retained in the depot for the same ablations depicted in Figure 6B. No significant differences in retention were observed, indicating that the infusion rate did not significantly influence retention, regardless of the needle gauge for small infusion volumes. Figure 6D shows box plots for the aspect ratios achieved for the same dataset. Consistent with the results of Exp. 1 and Exp. 2, no significant differences were observed for either a 10 mL/h infusion rate or manual infusion, regardless of the needle gauge, indicating that the needle gauge did not have a significant influence over the distribution shape. There is a larger range of aspect ratios (n.s.) for the 10 mL/h ablations, indicating that manual infusion may achieve more reliable and predictable distribution shapes.

## 4. Discussion

ECE ablation has the potential to be a low-cost alternative to thermal ablation for settings that lack access to thermal ablation and for tumors located in areas not amenable to thermal ablation. For ECE ablation to be translated to a clinical setting a full understanding of the multivariate influence of injection parameters on the resultant ablation volume and shape is necessary. This study examined the influence of multiple injection parameters (EC concentration, infusion volume, needle gauge, and infusion rate) on the resultant force, distribution volume, and shape. The experiments detailed here were not aimed at finding the optimal conditions for ablation. Rather, we focused upon the biophysical relationship between ECE injection parameters and the resultant distribution of ECE in tissue, as visualized with CT imaging.

The force studies, while limited in their conclusions due to only being collected into air, showed the high variability in force during an ECE injection, especially at higher ECE concentrations and using smaller needle diameters. ECE concentration was the largest contributor, as determined by the sum of squares, compared to needle gauge. The 12% ECE injections required force nearly four times higher than 6% ECE injected with the same needle gauge and a 27 G needle required force about three times as high as a 22 G when injecting the same ECE concentration. Combinations of high ECE concentrations and small needle gauges might not be feasible as manual injections due to the infusion force required.

Some volumes did vary significantly from each other; however, this is likely due to the higher variability seen in the longer injection times. The high variability in force seen in these injections could help explain the variability in injection distribution. We previously found that keeping injection pressures below a critical pressure helped reduced cracking in tissue [44]. The force measurement setup used is accurate for such a viscous substance but will struggle in cases where the force plateau is not reached due to a short injection time. Future studies will need to be conducted in tissue to investigate how these high forces impact the back pressure of different tissue types.

The 12% ECE achieved a superior distribution volume and retention compared to 6% ECE and exhibited a wider range of aspect ratio values across distribution volumes. We found that increasing the infusion volume had a greater impact at the higher ECE concentration. Further, 12% ECE had a stronger positive correlation with depot volume and infusion volume than did 6% ECE, but they produced similar results at smaller volumes. When selecting a polymer concentration for treatment, lower concentrations like 6% ECE may achieve more predictable ablation shapes and more comparable distribution volumes to 12% ECE for smaller infusion volumes, like 0.5 mL. However, if larger infusion volumes are needed, higher concentrations, like 12% ECE, may be preferable to achieve better retention and coverage. Studies of traditional ethanol ablation found similar positive correlation and variability in its resultant distribution, as measured by the radius of necrosis measured from cross sections of excised tissue [48], which may be a less accurate representation of ablation volume compared to CT, which gives a full three-dimensional rendering.

Interestingly, neither needle gauge nor infusion rate showed a significant influence on depot volume or retention. Thus, for our ablative therapy, the choice in needle may be left up to the physician or availability of supplies. A wider range in aspect ratio values was observed for the smaller needle (27 G) with the 12% ECE, likely resulting from the higher pressures needed to push a more viscous fluid through the smaller needle. Few studies have examined the effect of needle gauge on ablation distribution, but several studies evaluated the effect of needle gauge on the spread of analgesic blockades with mixed results: some studies reported that larger needles resulted in a higher level of spread [71] while others found that needle gauge did not influence spread [72]. It is likely that the results of ECE injections, including their variability, are dependent not only on the injectate’s physical properties, e.g., rheology, but also the anatomical location and biophysical properties of the tissues being injected. Clearly, follow-up examination of these additional parameters is necessary in various tissue types to better elucidate best practices for infusions depending on the treatment goals.

Lastly, infusion rate did not have a significant influence on depot volume or retention at smaller infusion volumes, suggesting that physicians could opt to use a controlled rate or manual infusion. Interestingly, this contrasts with results from studies of traditional ethanol ablation, which found that slower flow rates allowed for more spherical distribution of ethanol [64]. It is likely that our results differ from the conclusions of that study because of the EC polymer, which increases the injectate viscosity and undergoes a phase change upon injection into tissue. Ethanol alone has a viscosity close to water (1.2 cP), while the lowest ECE concentration used here had a viscosity of nearly 1000 cP before it underwent phase transition [35]. The EC polymer used has a strong impact on the conclusions drawn about the causative impacts of multiple injection parameters. Higher variability in distribution shape was observed for the 10 mL/h rate, likely due to dissipation of pressure during a manual injection, which is not possible when using an infusion pump.

We recognize several limitations with this work. First, we used ex vivo swine liver, which is highly vascular and impacts injection distributions in vivo and ex vivo. In vivo, vascular blood flow acts to carry away injection molecules that enter blood vessels. Ex vivo, the vasculature is collapsed, so fluid within it is essentially stationary. When the injectate material spreads into a vessel, the vessel provides a path with reduced resistance relative to the tissue, causing ready spread of the injectate through the vessels. While we accounted for this factor by analyzing the depot volume and using large sample sizes, it may not be appropriate to extrapolate these results to other tissues without further investigation. Second, high variability was observed for all depot volume and retention results. Again, due to the high vascularity of the liver, it was difficult to ensure consistent distance from vessels during needle placement. Future in vivo studies should use ultrasound or CT to guide needle placement and avoid large vasculature, as this would likely reduce the variance in the depot volume and retention. Finally, force studies conducted only allowed for the force of ECE through a syringe and needle to be characterized. Future studies will need to repeat measurements in tissue to understand its contribution. Conducting the studies into tissue could help to connect the variability seen in force to the variability seen in injection distribution.

Although the liver is commonly used for the assessment of the distribution and efficacy of ablative therapies [48,49,50,51,52,53,54,55,56], we plan to expand this work in the future to include other healthy tissues as well as cancerous lesions. Future studies should elucidate the effect of tissue biophysical differences (i.e., vascularity, stiffness, density). The liver is extremely vascular [73] and has low stiffness [74], therefore comparison to other tissues of lower vascularity and higher stiffness, more similar to tumors, is necessary to inform parameter selection for the treatment of larger lesions or tumors.

## 5. Conclusions

These experiments investigated the influence of various parameters (infusion force, infusion volume, EC concentration, needle gauge, and infusion rate) to determine relationships between the force required for infusion, resultant depot volume, retention, and shape. This study can serve as the basis for the future creation of a predictive computational model that uses tumor biophysical properties as inputs to determine optimal injection parameters (infusion volume, infusion rate, and needle gauge). This would not only improve the overall injection optimization process, but also objectively account for the interacting roles of the many formulation and injection characteristics that govern injection outcomes. Thus, there are tradeoffs when making decisions for ECE delivery: for smaller infusion volumes, the ECE concentration has little impact, while for larger infusion volumes, higher EC concentrations are necessary. While needle gauge has little impact on depot volume or retention, the use of a smaller needle with higher EC concentrations could reduce the predictability of the depot shape. Due to the high forces observed for 12% ECE, feasible flow rates for certain injection parameter combinations could be limited. Therefore, it is important to consider all parameters, the available supplies, as well as the tumor’s anatomical position and shape, to select delivery parameters to meet the ablation goals.

## Figures and Tables

**Table 1:** Summary of experiments and key parameters varied in each experiment.

Experiment	Parameters Assessed (*n* > 6 for Each Group)				
	Infusion Volume (mL)	EC Concentration (%)	Needle Gauge (G)	Needle Length * (inches)	Infusion Rate (mL/h)
1	0.5, 1, 1.5, 2, 2.5	6	27	0.5	10
		12			
2	0.5, 1, 1.5, 2, 2.5	6	22	0.75	10
		12			
3	0.5, 1	12	27	0.5	Manual Infusions
			22	0.75	

* Needle length varied due to manufacturer availability; however, all needles were inserted 0.5 inches into the tissue for all injections.

## Data Availability

All data supporting the reported results are available upon request from the authors.

## Supplementary Materials

The following supporting information can be downloaded at: https://www.mdpi.com/article/10.3390/polym16070997/s1, Figure S1: Representative 3D segmentations of the depot volume with and without non-depot injection spread; Figure S2: Representative 3D segmentations including non-depot injection spread for infusions using a 10 mL/h infusion rate; Figure S3: Representative 3D segmentations including non-depot injection spread for infusions performed manually.
