# Supplementary material for: Determining the Relationship between Delivery Parameters and Ablation Distribution for Novel Gel Ethanol Percutaneous Therapy in Ex Vivo Swine Liver
Source: Polymers (Basel). 2024 Apr 5;16(7):997. doi: 10.3390/polym16070997 (PMC11013462; doi:10.3390/polym16070997)
Supplement: Supplementary file 1 [file polymers-16-00997-s001.zip › polymers-2919331-supplementary.pdf]

Article

Determining the Relationship between Delivery Parameters and Ablation Distribution for Novel Gel Ethanol Percutaneous Therapy in Ex Vivo Swine Liver

Erika Chelales [1,*], Katriana von Windheim [1], Arshbir Singh Banipal [1], Elizabeth Siebeneck [1], Claire Benham [1], Corrine A. Nief [1], Brian Crouch [1], Jeffrey I. Everitt [2], Alan Alper Sag [3], David F. Katz [1] and Nirmala Ramanujam [1]

[1] Department of Biomedical Engineering, Duke University, Durham, NC 27708, USA; katriana.von.windheim@duke.edu (K.v.W.); arshbir.banipal@duke.edu (A.S.B.); cnief@stanford.edu (C.A.N.)
[2] Department of Pathology, Duke University Medical Center, Durham, NC 27710, USA; jeffrey.everitt@duke.edu
[3] Department of Radiology, Division of Vascular and Interventional Radiology, Duke University Medical Center, Durham, NC 27710, USA
* Correspondence: erika.chelales@duke.edu

Supplemental Figures

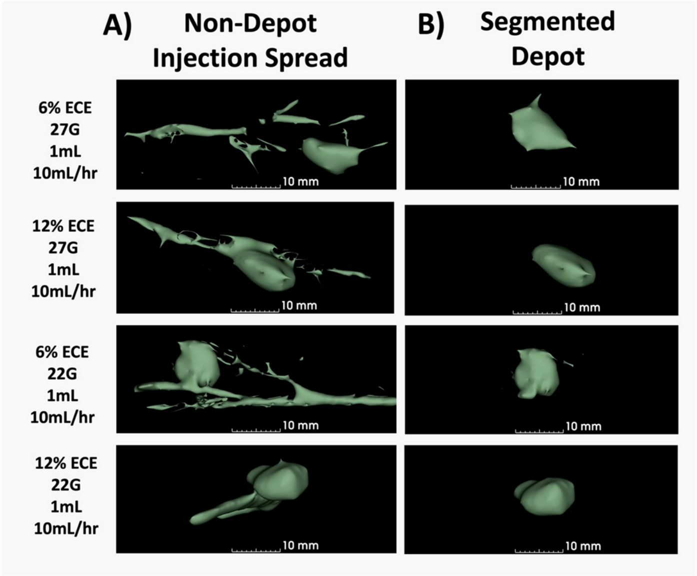

Figure S1. Representative 3D segmentations of the depot volume with and without non-depot injection spread. A) Representative 3D segmentations of the non-depot injection spread for 1mL infusions at 10mL/hr. with 27G and 22G needles. The 3D segmentations show non-depot spread of the injectate into nearby vasculature. B) Representative 3D segmentations of the depot for 1mL infusions at 10mL/hr. with 27G and 22G needles. The non-depot spread into nearby collapsed vasculature is removed to create the 3D segmentation of the depot.

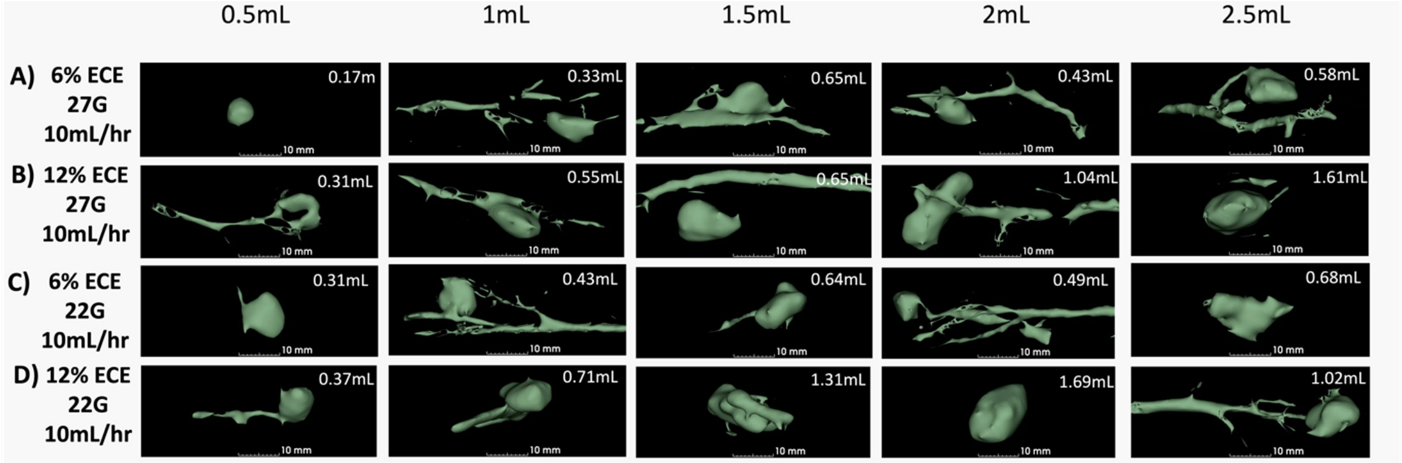

Figure S2. Representative 3D segmentations including non-depot injection spread for infusions using a 10mL/hr. infusion rate. A-D) Representative 3D segmentations including non-depot injection spread for 0.5mL, 1mL, 1.5mL, 2mL, and 2.5mL infusion volumes for 6% ECE infused with a 27G needle (A), 12% ECE infused with a 27G needle (B), 6% ECE infused with a 22G needle (C), and 12% ECE infused with a 22G needle (D). All infusions were performed with a 10mL/hr. infusion rate.

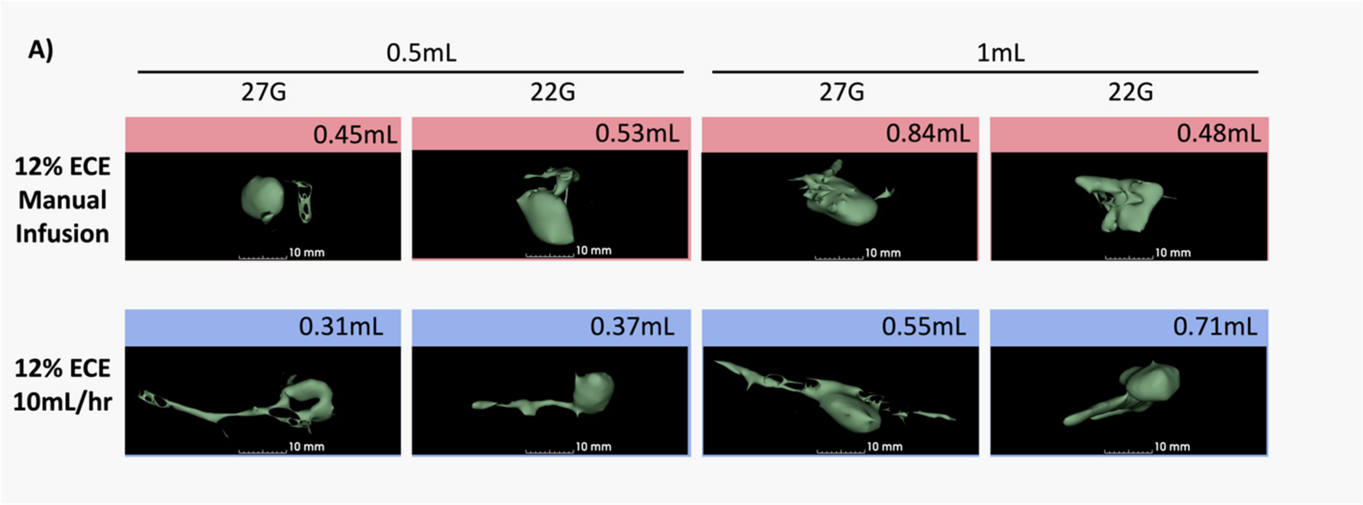

Figure S3. Representative 3D segmentations including non-depot injection spread for infusions performed manually. A) Representative 3D segmentations including non-depot injection spread for 0.5mL and 1mL infusion volumes for 12% ECE using both 22G and 27G needles and 10mL/hr. and manual infusion rates.
